# Supplementary material for: The prognostic significance of additional localized treatment to primary lesion in patients undergoing hormone therapy for metastatic hormone-sensitive prostate cancer: A systematic review and meta-analysis
Source: PLoS One. 2024 Jun 10;19(6):e0304963. doi: 10.1371/journal.pone.0304963 (PMC11164370; doi:10.1371/journal.pone.0304963)
Supplement: S1 Fig — RCTs: randomized controlled studies. (DOCX) [file pone.0304963.s001.docx]

|  | **Random sequence generation** | **Allocation concealment** | **Blinding of participants** | **Blinding of outcome** | **Incomplete outcome data** | **Selective reporting** | **Other Bias** |
| --- | --- | --- | --- | --- | --- | --- | --- |
| **Study and year** | **Assessment of the main biases** | | | | | | |
| Parker, 2018 |  |  |  |  |  |  |  |
| Boeve, 2019 |  |  |  |  |  |  |  |
| Dai, 2022 |  |  |  |  |  |  |  |

**Figure S1.** Risk of bias assessment of the included RC
