# Supplementary material for: The prognostic significance of additional localized treatment to primary lesion in patients undergoing hormone therapy for metastatic hormone-sensitive prostate cancer: A systematic review and meta-analysis
Source: PLoS One. 2024 Jun 10;19(6):e0304963. doi: 10.1371/journal.pone.0304963 (PMC11164370; doi:10.1371/journal.pone.0304963)
Supplement: Attachment — Submitted filename: english proofreading.pdf [file pone.0304963.s004.pdf]

2-21-1 Tsuruya-cho, Kanagawa-Ku, Yokohama, Kanagawa Pref. 221-0835 Japan
Tel: 045-290-7200 Fax: 045-290-7201
E-Mail: info@nai.co.jp

2024/05/15

To Whom It May Concern:

An experienced editor whose first language is English has carefully proofread this paper entitled:

The prognostic significance of additional localized treatment to primary lesion in patients undergoing hormone therapy for metastatic hormone-sensitive prostate cancer: a systematic review and meta-analysis

Our company specializes in editing papers written by physicians and scientists whose native language is not English.

Shuji Ito
President NAI, Inc.
